# Exposure to cadmium and lead is associated with diabetic kidney disease in diabetic patients

**DOI:** 10.1186/s12940-023-01045-z

**Published:** 2024-01-03

**Authors:** Yuan Zhang, Xiaoyu Gong, Runhong Li, Wenhui Gao, Daibao Hu, Xiaoting Yi, Yang Liu, Jiaxin Fang, Jinang Shao, Yanan Ma, Lina Jin

**Affiliations:** 1 Department of Epidemiology and Biostatistics, School of Public Health, Jilin University, No.1163 Xinmin Street, Changchun, Jilin 130021 P.R. China; 2 Department of Biostatistics and Epidemiology, School of Public Health, China Medical University, No. 77 Puhe Road, Shenyang North New Area, Shenyang, Liaoning Province 110122 P.R. China; 3 Department of Public Health, Xinjiang Medical University, Urumqi, 830011 P.R. China

**Keywords:** Blood cadmium, Blood lead, Urinary cadmium, Urinary lead, Diabetic kidney disease

## Abstract

**Background:**

Cadmium (Cd) and lead (Pb) exhibit nephrotoxic activity and may accelerate kidney disease complications in diabetic patients, but studies investigating the relation to diabetic kidney disease (DKD) have been limited. We aimed to examine the associations of Cd and Pb with DKD in diabetic patients.

**Methods:**

3763 adults with blood metal measurements and 1604 adults with urinary ones who were diabetic from National Health and Nutrition Examination Survey (NHANES) 2007–2016 were involved. Multivariate logistic regression models were used to analyze the associations of blood Cd (BCd), blood Pb (BPb), urinary Cd (UCd), and urinary Pb (UPb) with DKD.

**Results:**

BPb, BCd, and UCd levels were higher among participants with DKD than diabetics without nephropathy, but UPb performed the opposite result. BPb and UCd were significantly associated with DKD in the adjusted models (aOR, 1.17 (1.06, 1.29);1.52 (1.06, 2.02)). Participants in the 2nd and 3rd tertiles of BPb and BCd levels had higher odds of DKD, with a significant trend across tertiles, respectively (all *P*-trend < 0.005). Multiplication interaction was also identified for BPb and BCd (*P* for interaction = 0.044).

**Conclusion:**

BPb, BCd, and UCd were positively associated with the risk of DKD among diabetic patients. Furthermore, there were the dose-response relationship and multiplication interaction in the associations of BPb, BCd with DKD.

**Supplementary Information:**

The online version contains supplementary material available at 10.1186/s12940-023-01045-z.

## Background

The increasing worldwide prevalence of diabetes and chronic kidney disease (CKD) has prompted greater attention and efforts to address diabetic kidney disease (DKD, also known as diabetic nephropathy), which is a growing epidemic [1]. There were an estimated 425 million cases of diabetes among adults worldwide in 2017 [2]. The prevalence of CKD has been relatively steady at under 15% among adults for the last 15 years in the United States, according to recent data from the National Health and Nutrition Examination Survey (NHANES) [3]. In China, the burden of diabetes continues to rise, with a projected prevalence of 9.7 percent by 2030 [4]. The Global Burden of Disease Study data shows that in 2019, the death cases of CKD were 197 thousands in China, of which 76 thousands deaths were due to diabetes-related CKD, accounting for 38.58% of the total [5, 6]. It was reported that half of the diabetic patients had diabetic microvascular complications (DKD, diabetic retinopathy, and neuropathy) [7], and DKD can be found in 31.1% of diabetic patients [8]. The all-age mortality rate attributed to DKD rose by one-third, increasing from 4.5 per 100,000 in 1990 to 6.0 per 100,000 in 2016 in China [9]. DKD is the strongest predictor of morbidity and premature mortality in individuals with diabetes [10], carrying enormous disease and financial burden. Hence, the primary objectives in the lifelong management of diabetes involve the prevention and treatment of associated complications.

Cadmium (Cd) and lead (Pb) show nephrotoxic activity as environmental pollutants [11, 12], Evidence suggests that diabetic patients are more susceptible to renal toxic effects of Cd and Pb [13]. The association of Cd exposure at a high level with nephrotoxic effects is remarkable [14]. However, some studies on the relationship between Cd exposure and population health showed that untoward effects might also generate at lower exposure levels [15–17]. The half-life of Cd in the kidney is 10–30 years, and the body burden of Cd influences the urinary Cd concentration. Thus, urinary Cd (UCd) exhibits recent and past exposure, whereas blood Cd (BCd) only exhibits recent exposure. For Pb, the kidney is the central part of the target organs [18]. Evidence showed that even at low levels, Pb has adverse health effects on children and adults [19]. The most widely used biomarker of Pb exposure is blood Pb (BPb), which exhibits whole-body burden and more recent exposure to Pb [18, 20]. Pb in the urinary (UPb) can be helpful when collected for long-term biomonitoring, which reflects the amount of Pb that has diffused from the plasma and excreted through the kidneys [21].

Although from 1999 to 2016, Americans experienced a dramatic decrease in Pb and Cd levels in their bodies, the ideal heavy metal content in the human body is 0 [22, 23]. In available studies based on NHANES, the relationship of urinary and/or blood Pb and/or Cd with diabetes among the general population has been well established [24, 25]. Some epidemiological studies have investigated that urinary and/or blood Pb and/or Cd exposure is associated with renal dysfunction and/or CKD in the general population [21, 26–29], prompting that exposure to Pb and/or Cd may accelerate kidney disease complications in diabetics patients, however, epidemiological studies on DKD among diabetics patients have been very limited. While one cross-sectional study revealed the positive association between BPb and DKD among diabetic patients in China [30], few studies have focused on independent and combined associations of urinary and blood Pb and Cd with DKD among diabetic patients in the United States. In this cross-sectional study involving a substantial sample of diabetic patients in the United States, we examined the associations of urinary and blood Pb and Cd, separately and jointly, with the presence of DKD.

## Materials and methods

### Study population

NHANES program is designed to assess the health and nutritional status of the non-institutionalized civilian population in the United States (https://www.cdc.gov/nchs/nhanes/about_nhanes.htm). Two samples were used for this analysis: (A) samples with Cd and Pb in the blood samples of the diabetic, (B) samples with Cd and Pb in the urinary samples of the diabetic, which included 3763 and 1604 participants, with complete data on the outcomes, without pregnancy. See details in Figure S1.

### Measurements and variables

Diabetes was defined as “yes” and “no” based on questionnaire and laboratory data, and kidney disease was defined as albuminuria and/or the estimated glomerular filtration rate (eGFR) < 60 mL/min/1.73 m^2^ [31].

BPb, BCd, UPb, and UCd were tested by inductively coupled plasma-dynamic reaction mass spectrometry (ICP-DRC-MS). Values of concentrations below the limit of detection (LOD) were imputed values of LOD/$\surd 2$. Detailed information on laboratory quality assurance and monitoring is available at https://www.cdc.gov/nchs/nhanes/index.htm.

All statistical models were adjusted for age (<60 years, ≥ 60 years), sex (male, female), race/ethnicity (non-Hispanic White, all others), education (high school and under, high school above), PIR (poverty-income ratio; <1, ≥ 1), BMI (Body Mass Index; < 30 kg /m^2^, ≥ 30 kg /m^2^), smoking status (smokers, serum cotinine > 14 ng/mL; non-smokers, serum cotinine ≤ 14 ng/mL), alcohol consumption (drinker, > 30 g/d for male, > 20 g/d for female; non-drinker, ≤ 30 g/d for male, ≤ 20 g/d for female, according to the first of two 24-hour dietary recall interviews), fish eaten during the past 30 days (no, yes), physical activity (PA, meeting and not meeting PA Guidelines, according to the 2018 PA Guidelines), and hypertension (no, yes) (Supplementary methods).

### Statistical analysis

The NHANES uses design weighting to ensure the representativeness of the data. These weights were applied in the analyses. Counts and percentages were used for categorical variables, compared using the Rao-Scott-χ^2^ test, and medians and interquartile ranges were used for measurement variables, compared using a t-test. Multivariate logistic regression models were used to estimate the associations of Pb and Cd with DKD, adjusted for age, sex, race/ethnicity, education, PIR, BMI, smoking status, alcohol consumption, fish eaten during the past 30 days, physical activity, and hypertension. Stratification analysis by covariates was also performed. Multiplication interactions were used to explore whether there were interactions between Pb, Cd, and DKD. All analyses were conducted using IBM SPSS software version 24.0 and R version 4.2.1, with the statistical significance level set at a 2-sided *P* < 0.05. Forest plots of *P* for interaction were implemented with the R package “forestploter”.

## Results

### Characteristics of participants

Table 1 provided the characteristics of sociodemographics and biochemistry of the participants by diabetes with and without nephropathy. BPb and BCd levels were higher for subjects with DKD than for subjects with diabetes without nephropathy (BPb levels, 1.38 (0.93, 2.10) vs. 1.06 (0.74, 1.64)), respectively; BCd levels, 0.37 (0.22, 0.61) vs. 0.30 (0.18, 0.51), respectively). A similar trend was observed for UCd (0.30 (0.16, 0.54) vs. 0.25 (0.14, 0.46)). For more information, see Table S1.

**Table 1:** Characteristics of sociodemographic and biochemistry in participants^a^

Variables	In blood (n = 3763)		*P*	In urine (n = 1604)		*P*
	Diabetes without nephropathy (n = 2272)	Diabetes nephropathy (n = 1491)		Diabetes without nephropathy (n = 981)	Diabetes nephropathy (n = 623)	
Age (years)			**< 0.001**			**< 0.001**
< 60	1126 (58.3)	379 (29.7)		488 (58.4)	158 (29.8)	
≥ 60	1146 (41.7)	1112 (70.3)		493 (41.6)	465 (70.2)	
Gender			0.554			0.609
Male	1173 (53.2)	799 (51.7)		507 (52.5)	341 (50.5)	
Female	1099 (46.8)	692 (48.3)		474 (47.5)	282 (49.5)	
Race/Hispanic			0.521			0.609
Non-Hispanic White	757 (60.3)	575 (61.4)		315 (60.4)	231 (62.1)	
All others	1515 (39.7)	916 (38.6)		666 (39.6)	392 (37.9)	
Education			**0.001**			**0.012**
≤high school	1305 (46.8)	949 (56.6)		569 (47.6)	403 (56.9)	
>high school	963 (53.2)	539 (43.4)		411 (52.4)	218 (43.1)	
PIR			**0.002**			**0.002**
< 1	475 (15.4)	363 (20.1)		196 (14.5)	164 (22.4)	
≥ 1	1569 (84.6)	976 (79.9)		680 (85.5)	393 (77.6)	
BMI (kg/m^2^)			0.429			0.975
< 30	948 (38.8)	591 (36.9)		391 (38.0)	253 (38.1)	
≥ 30	1287 (61.8)	850 (63.1)		576 (62.0)	352 (61.9)	
Physical activity			**< 0.001**			**< 0.001**
Not meeting PA Guidelines	1201 (50.0)	949 (62.7)		501 (47.1)	394 (62.1)	
Meeting PA Guidelines	1065 (50.0)	536 (37.3)		477 (52.9)	223 (37.9)	
Smoking status			**0.047**			**0.044**
Non-smoker	1730 (76.5)	1192 (81.0)		727 (76.1)	479 (82.5)	
Smoker	488 (23.5)	282 (19.0)		202 (23.9)	114 (17.5)	
Alcohol consumption			**0.020**			**0.010**
Non-drinker	1930 (93.4)	1298 (93.4)		826 (89.5)	539 (94.9)	
Drinker	182 (10.1)	79 (6.6)		84 (10.5)	29 (5.1)	
Fish eaten			0.940			0.230
Not eating fish	549 (25.0)	361 (24.5)		223 (23.5)	158 (26.7)	
Eating fish	1565 (74.7)	1018 (73.9)		688 (76.3)	410 (71.0)	
Hypertension			**< 0.001**			**< 0.001**
No	865 (41.2)	308 (22.2)		382 (41.4)	126 (23.6)	
Yes	1368 (58.8)	1167 (77.8)		589 (58.6)	490 (76.4)	
Pb (ug/dL)	1.06 (0.74, 1.64)	1.38 (0.93, 2.10)	0.055	0.40 (0.23, 0.70)	0.37 (0.25, 0.66)	0.475
Cd (ug/L)	0.30 (0.18, 0.51)	0.37 (0.22, 0.61)	0.066	0.25 (0.14, 0.46)	0.30 (0.16, 0.54)	0.130

^a^Characteristics of participants are given as frequency (weighted percentage) in each category or median (interquartile range) in each continuous variable

Bold text indicates statistical significance

### Differences in associations of BPb, BCd, UPb, and UCd with DKD

Analysis in metals exposure as continuous variables showed statistically significant associations of BPb, and UCd with DKD after adjusting for covariates (Model 3: 1.17 (1.06, 1.29); 1.52 (1.06, 2.02)). At the same time, the statistically significant increase in the strength of associations moving from the lowest tertile (T1) to the highest tertile (T3) of BPb and BCd levels indicated evidence of dose-response (*P*-trend < 0.05). Compared with the lowest tertile (T1), participants with higher BPb and BCd levels (T3) had a 95% and 68% higher risk of DKD. (Model 3: 1.95 (1.42, 2.67); 1.68 (1.26, 2.23)) (Table 2).

**Table 2:** Associations of metals exposure with diabetic kidney disease in diabetic patients

	Metals exposure (in blood)			Metals exposure (in urine)		
	Model 1	Model 2	Model 3	Model 1	Model 2	Model 3
	OR (95%CI)	OR (95%CI)	OR (95%CI)	OR (95%CI)	OR (95%CI)	OR (95%CI)
**Pb** ^**a**^						
T1	1.00 (Reference)	1.00 (Reference)	1.00 (Reference)	1.00 (Reference)	1.00 (Reference)	1.00 (Reference)
T2	**1.56 (1.25, 1.94)**	**1.45 (1.15, 1.82)**	**1.43 (1.09, 1.86)**	1.23 (0.90, 1.70)	1.15 (0.78, 1.67)	1.05 (0.67, 1.63)
T3	**2.34 (1.83, 2.97)**	**1.93 (1.49, 2.51)**	**1.95 (1.42, 2.67)**	1.03 (0.74, 1.43)	0.95 (0.66, 1.36)	0.88 (0.55, 1.42)
P for trend	**< 0.001**	**< 0.001**	**< 0.001**	0.957	0.702	0.657
Per 1 change	**1.25 (1.12, 1.40)**	**1.16 (1.06, 1.28)**	**1.17 (1.06, 1.29)**	0.91 (0.75, 1.11)	0.90 (0.74, 1.09)	0.88 (0.71, 1.11)
**Cd** ^**b**^						
T1	1.00 (Reference)	1.00 (Reference)	1.00 (Reference)	1.00 (Reference)	1.00 (Reference)	1.00 (Reference)
T2	**1.48 (1.20, 1.82)**	1.24 (0.97, 1.57)	**1.32 (1.03, 1.69)**	1.21 (0.87, 1.68)	1.03 (0.73, 1.46)	1.07 (0.71, 1.63)
T3	**1.70 (1.38, 2.09)**	**1.47 (1.16, 1.85)**	**1.68 (1.26, 2.23)**	1.40 (0.99, 1.98)	1.23 (0.86, 1.77)	1.44 (0.90, 2.32)
P for trend	**< 0.001**	**0.003**	**0.002**	0.070	0.234	0.147
Per 1 change	**1.17 (1.01, 1.34)**	**1.18 (1.02, 1.37)**	1.26 (0.97, 1.65)	**1.34 (1.03, 1.74)**	**1.30 (1.01, 1.68)**	**1.52 (1.06, 2.02)**

Model 1: unadjusted

Model 2: adjusted for age, sex, race/ethnicity, education, PIR, BMI

Model 3: Model 2 + PA, smoking status, alcohol consumption, fish eaten, hypertension. Lead and cadmium were mutually adjusted

*Abbreviations*: OR, odds ratio; CI, confidence interval; Pb, lead; Cd, Cadmium; PIR, poverty-income ratio; BMI, body mass index; PA, physical activity

^a^Pb, BPb, ug/dL, UPb, ug/L; ^b^ Cd, BCd, ug/L, UCd, ug/L

Bold text indicates statistical significance

### Interaction of Cd and Pb co-exposure on the presence of DKD

**Fig. 1:**
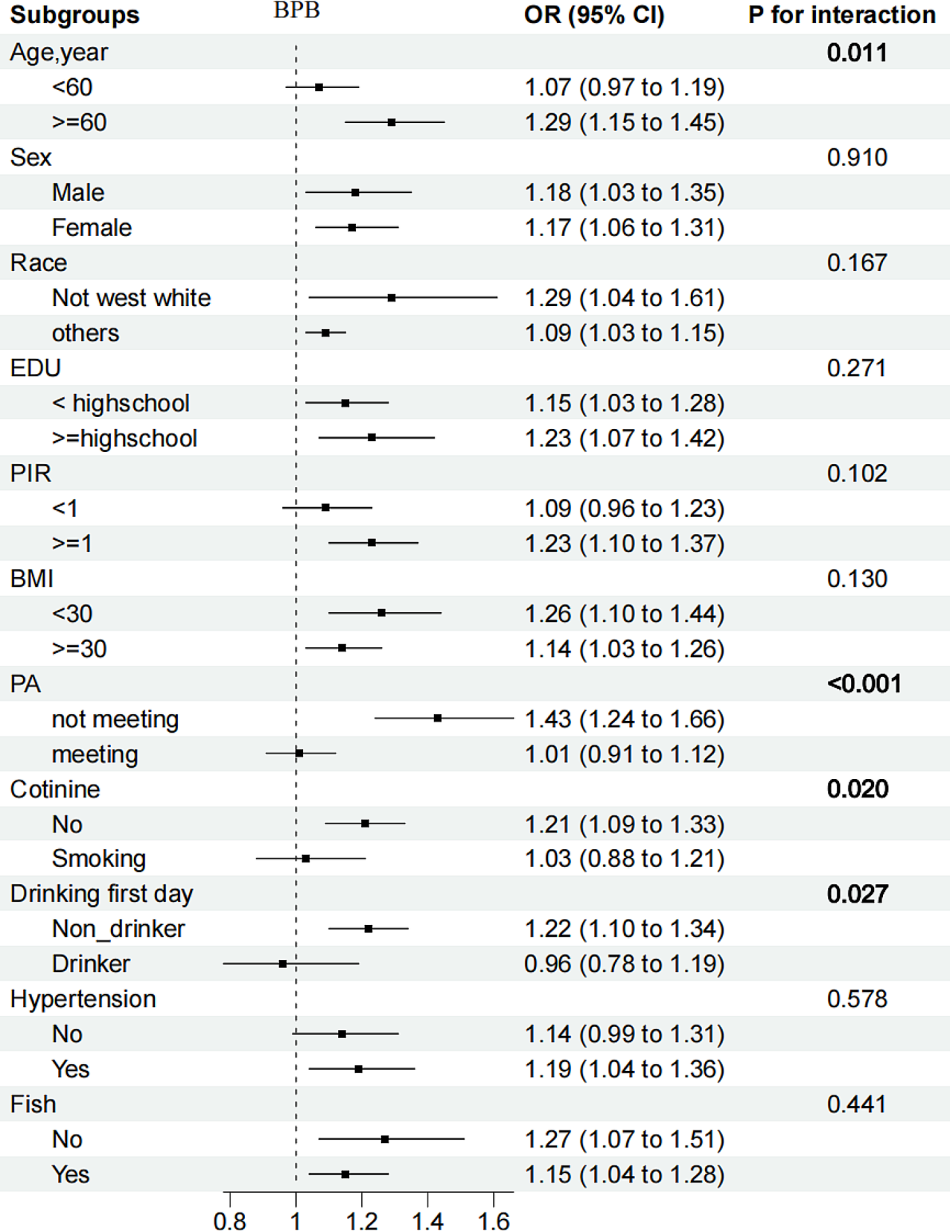
Stratified OR and 95% CIs for BPb and DKD. Weighted logistic models were used in the analysis. Models were adjusted for age, sex, race/ethnicity, education, PIR, BMI, smoking status, alcohol consumption, fish eaten during the past 30 days, physical activity, and hypertension. Of note, the variables examined in this table were not adjusted

**Fig. 2:**
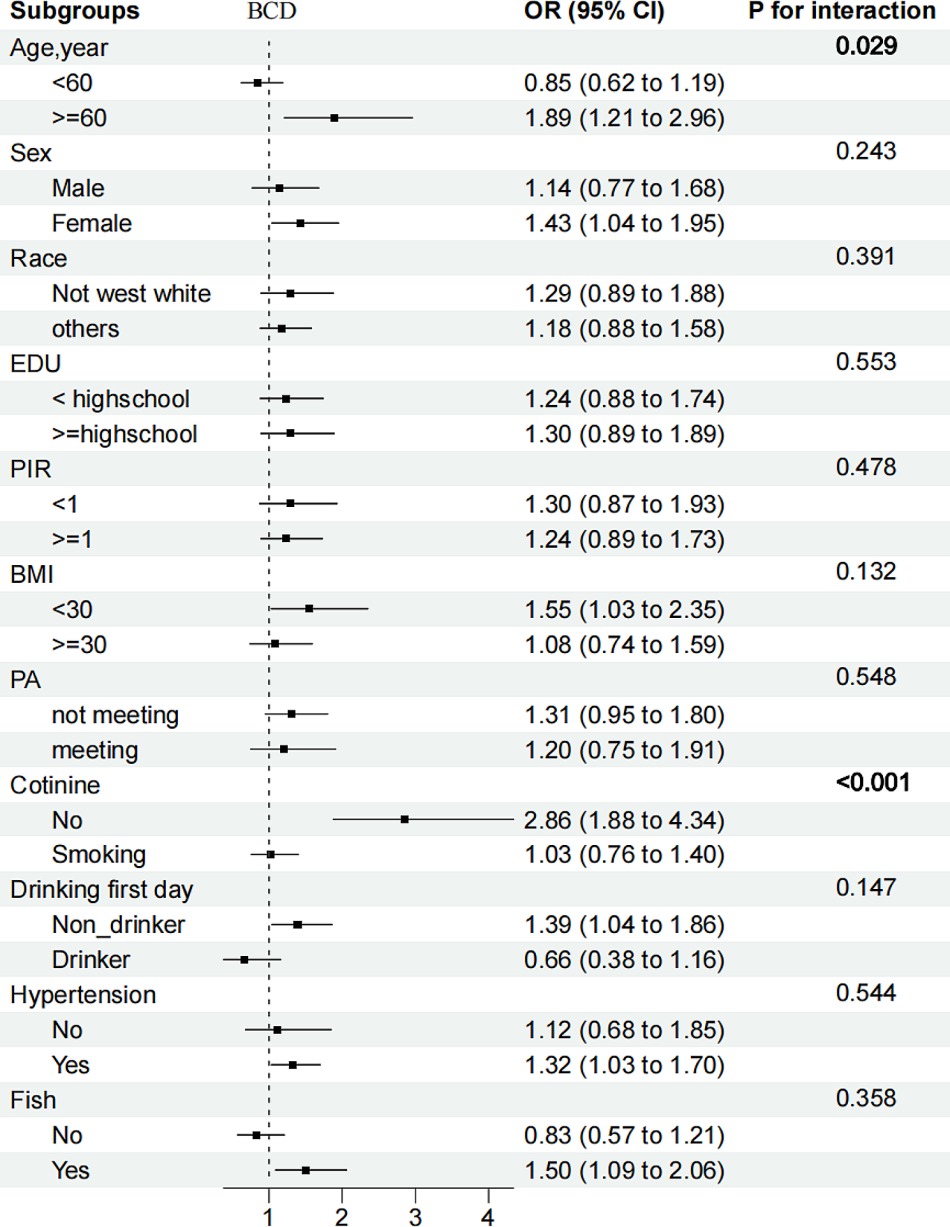
Stratified OR and 95% CIs for BCd and DKD. Weighted logistic models were used in the analysis. Models were adjusted for age, sex, race/ethnicity, education, PIR, BMI, smoking status, alcohol consumption, fish eaten during the past 30 days, physical activity, and hypertension. Of note, the variables examined in this table were not adjusted

**Fig. 3:**
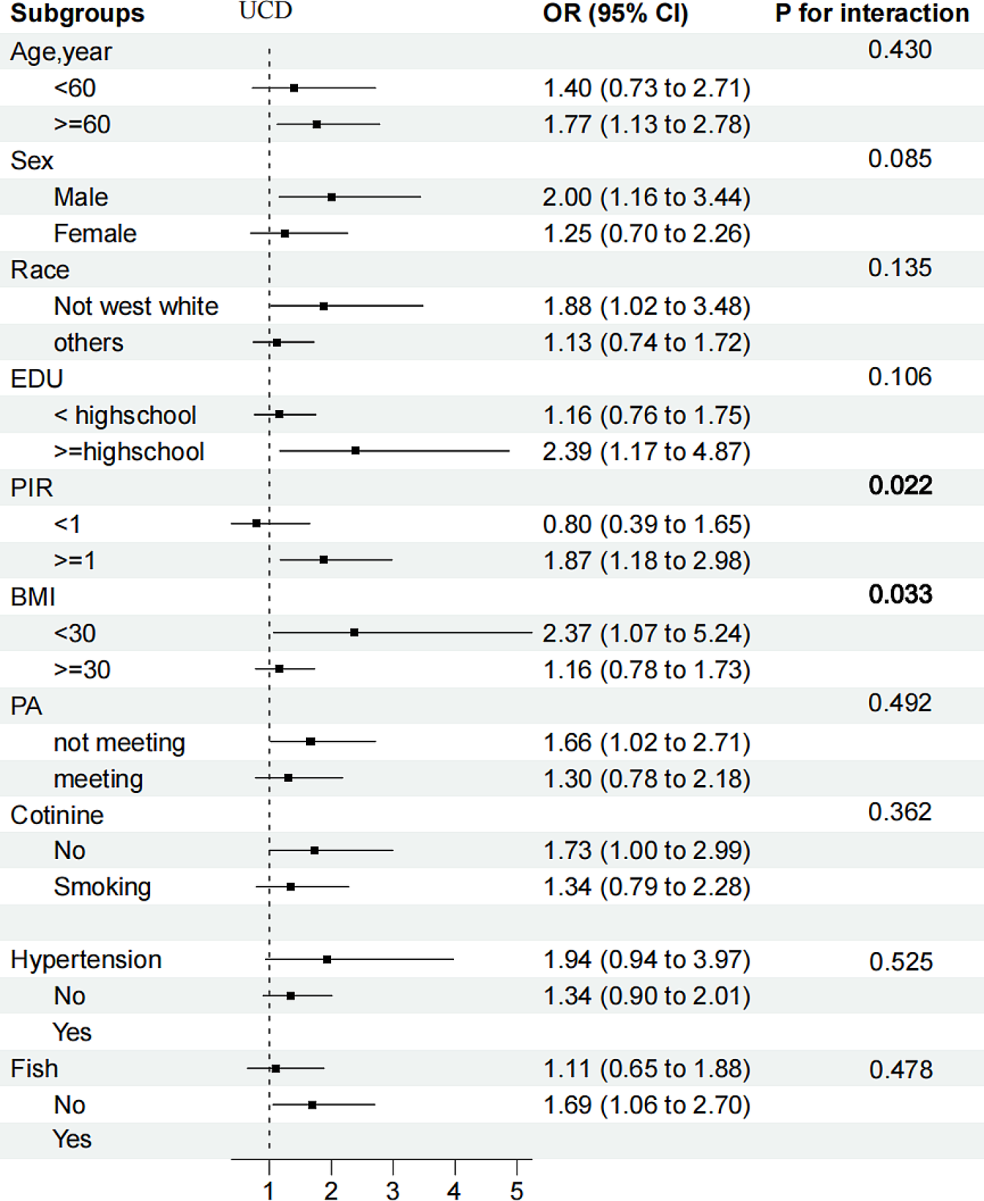
Stratified OR and 95% CIs for UCd and DKD. Weighted logistic models were used in the analysis. Models were adjusted for age, sex, race/ethnicity, education, PIR, BMI, smoking status, alcohol consumption, fish eaten during the past 30 days, physical activity, and hypertension. Of note, the variables examined in this table were not adjusted

Associations of high BPb + low BCd (OR: 1.90 (1.35, 2.68)), low BPb + high BCd (OR: 1.51 (1.10, 2.08)), and high BPb + high BCd (OR: 2.58 (1.93, 3.45)), with DKD were all positive, with low BPb + low BCd as a reference after adjusting for covariates (*P* for interaction = 0.044), supporting the existence of the multiplication interaction (Table 3). Risk-group distribution was defined as low UPb + high UCd compared with the reference group (OR: 1.91 (1.21, 3.02)), and no statistically significant interaction was observed between UPb and UCd (*P* for interaction = 0.071). In subgroup analysis, we observed consistent associations between the heavy metals and DKD across subpopulations. (Figures 1, 2, and 3).

**Table 3:** Multiplication interaction of Pb and Cd with DKD

Levels of Metals	N	OR (95%CI)	*P* for interaction
Metals in blood			**0.044**
Low BPb + Low BCd	916	1.00 (reference)	
High BPb + Low BCd	763	**1.90 (1.35, 2.68)**	
Low BPb + High BCd	702	**1.51 (1.10, 2.08)**	
High BPb + High BCd	1382	**2.58 (1.93, 3.45)**	
Metals in urine			0.071
Low UPb + Low UCd	240	1.00 (reference)	
High UPb + Low UCd	288	1.20 (0.76, 1.88)	
Low UPb + High UCd	219	**1.91 (1.21, 3.02)**	
High UPb + High UCd	637	1.29 (0.88, 1.88)	

*Note*: P50 was set as the cut-off point, and BPb, BCd, UPb, and UCd were divided into high and low exposure groups, 1 was high exposure, 0 was low exposure, and the relative risk (relative risk, RR) was replaced by the OR value calculated by the logistic regression model

Bold text indicates statistical significance

## Discussion

In this study, we found BPb, BCd and UCd were positively significantly associated with the presence of DKD among diabetic patients in the United States. However, a correlation was not observed between UPb and DKD. Furthermore, we also found that the dose-response relationship could indicate exposures and outcomes of BPb and BCd. The multiplication interaction existed between BPb and BCd; no such results were seen for either UCd or UPb.

First, BPb and UCd were positively significantly associated with the presence of DKD in our findings. Moreover, we also found that participants in the second and third BPb tertile had statistically significantly higher odds than the referent BPb tertile; similar results were observed for tertiles in BCd, noting the presence of dose-response relationships. This is consistent with previous studies. Previous observational studies have suggested that the BPb were related to the renal function decline or kidney disease in the general population [32–34] and diabetic patients [13, 22, 30]. In two cross-sectional surveys in China among 720 participants and 747 participants, respectively, a significant positive correlation was found between Pb exposure and renal function [32, 34]. A cross-sectional survey which enrolled 2210 adults across twelve provinces in China suggested that association was found between Cd and CKD [33]. Also, it was previously suggested that greater BPb and BCd were associated with more significant harm to the kidney in the general population [29]. A previous cross-sectional study involving 3,473 middle-aged and elderly diabetic patients revealed a strong and dose dependent positive correlation between BPb and DKD. Patients in the fourth quartile of BPb concentration had a significantly higher odds of DKD compared to those in the first quartile (*P* for trend < 0.05) [30]. In an observational study among 4033 diabetic patients in China, higher blood lead levels were linearly, independently associated with higher urinary albumin-to-creatinine ratio and prevalence of albuminuria [22]. Furthermore, in a cohort study conducted in the Netherlands, this study found clear associations between these elements and albuminuria and reduced creatinine clearance, respectively, with concentrations of BCd and BPb considerably below the values for acute toxicity, among diabetic patients [13].

Furthermore, in this study, we observed the effects of combined exposure to Pb and Cd on DKD in US adults, we found the multiplication interaction between BPb and BCd, a highest correlation with DKD was shown when both BPb and BCd were at higher concentrations. To our knowledge it was not investigated in available research about DKD, this indicated the importance of simultaneous exposure to Pb and Cd for population health risk assessment. Based on the DKD definition, a randomized controlled trial from China found that subjects with higher BCd / BPb and UCd / UPb had a higher probability of developing renal tubular dysfunction [35]. The interaction between BPb and BCd was consistent with our study, we found whether low BPb and high BCd group, high BPb and low BCd group, or high BPb and high BCd group showed a higher DKD risk compared to low BPb and BCd group, with the high BPb and high BCd group having the highest risk. A similar trend was also observed in low UPb and high UCd, but with no statistical significance of the interaction. Despite evidence that high levels of Pb and Cd co-exposure were significantly correlated to an increase in FPG and odds of diabetes in Chinese adults [36]. We suggest the existence of interaction is more likely because Pb increases the impact of Cd exposure on early renal biomarkers, Pb and Cd co-exposure may increase the risk of renal tubular dysfunction than by Cd or Pb alone [35, 37]. The results were supported by an animal experiment; for the SD rats, Pb and Cd were practically additive-toxic [38]. Moreover, a statistically significant risk factor relationship was observed with BPb for both women and men, the association between BCd and DKD was evident in the case of accompanying females, but UCd was significantly associated with males. It might be explained by the fact that heavy metals are associated with sex hormones, and maternal Cd exposure can influence fetal in a sex-specific manner [25, 39]. These results suggested that intervention could be performed from the perspective of limiting exposure to heavy metals such as Pb and Cd, especially co-exposure, to reduce the possibility of concurrent renal damage in diabetic patients, which might have certain public health significance.

Potential biological mechanisms might explain why toxic heavy metals (Pb and Cd) induce DKD. Both long-term Pb stores and circulating Pb might cause renal function decline among middle-aged and elderly diabetics [22]. Similarly, metabolic changes in diabetes might increase susceptibility to Cd-induced kidney damage [40]. In general, the biological half-life of Cd is 10 to 30 years in the human renal cortex and 3–4 months in human blood. In cases of toxicity, the half-life of Pb in adults’ blood is estimated to be 28 to 36 days [41]. It suggests that the different correlations of different metals in blood and urine with DKD might be related to the half-life of the metals. Apart from this, lead or cadmium causes oxidative stress, resulting in increased reactive oxygen species, and impaired oxidant/antioxidant balance may be relevant to kidney injury induced by lead and cadmium [42]. In vivo, animal experiments suggested that combined exposure to Pb and Cd could escalate oxidative stress more than alone, possibly accounting for the multiplication interaction between BPb and BCd [43]. Nevertheless, the exact mechanisms require further study.

There are several strengths in the analysis. This study focused on exploring the associations of urinary and blood Pb and Cd with DKD among diabetic patients based on a large and nationally representative sample. Furthermore, biomonitoring measurements are the essential health assessment regarding exposure to metals, indicating all environmental sources combined with metals in people. This study also has limitations, such as this cross-sectional study could not adequately disentangle the complex interplay between DKD risk factors and hindered us from drawing inferences regarding the temporality of the associations; we could not prove a reverse causality. Second, the biomarkers assessed in this study were measured only at one point, and thus providing only a snapshot of exposures might only partially estimate participant exposure to these metals.

## Conclusion

Our findings showed that BCd, UCd, and BPb were positively associated with the risk of DKD among diabetic patients in the United States. Furthermore, the study also showed the dose-response relationship and the multiplication interaction between BPb, BCd, and DKD. In conclusion, this work provided insights into metal exposures in blood and urine and their interrelationships with DKD.

### Electronic supplementary material

Below is the link to the electronic supplementary material.

**Supplementary Material 1:** Supplementary Methods and supplemental Results

## Data Availability

The data supporting this study’s findings are available at https://www.cdc.gov/nchs/nhanes/about_nhanes.htm. Information from NHANES is made available through extensive publications and articles in scientific and technical journals. For data users and researchers worldwide, survey data and easy-to-use CD-ROMs are available on the internet.

## Glossary

CKD: chronic kidney disease.
DKD: diabetic kidney disease.
eGFR: estimated glomerular filtration rate.
NHANES: National Health and Nutrition Examination Survey.
Cd: cadmium.
Pb: lead.
UCd: urinary cadmium, or cadmium in urinary.
BCd: blood cadmium, or cadmium in blood.
UPb: urinary lead, or lead in urinary.
BPb: blood lead, or lead in blood.
aOR: adjusted odds ratio.
CI: confidence interval.
CDC: Centers for Disease Control and Prevention.
