# Supplementary material for: Exposure to cadmium and lead is associated with diabetic kidney disease in diabetic patients
Source: Environ Health. 2024 Jan 3;23:1. doi: 10.1186/s12940-023-01045-z (PMC10763104; doi:10.1186/s12940-023-01045-z)
Supplement: Supplementary file 1 — Supplementary Material 1: Supplementary Methods and supplemental Results [file 12940_2023_1045_MOESM1_ESM.docx]

**Supplementary Materials**

**Exposure to cadmium and lead is associated with diabetic kidney disease in diabetic patients**

**Appendix 1: Supplementary Methods**

**Appendix 2: Supplemental Results**

**Appendix 1**

**Supplementary Methods**

**2.1 Study population**

In this study, we used data from five NHANES survey cycles (2007–2008, 2009–2010, 2011–2012, 2013–2014, and 2015–2016) to investigate blood and urinary cadmium and lead and diabetic kidney disease.

**2.2 Measurements and variables**

Diabetes was defined as meeting any of the following conditions: 1) the “yes” answer to the questions: “Other than during pregnancy, have you ever been told by a doctor or health professional that you have diabetes or sugar diabetes?” or “now taking insulin” or “now taking diabetic pills”, or; 2) their hemoglobin A1c was greater than or equal to 6.5%. or; 3) their fasting (8–24 h) plasma glucose was greater than or equal to 7.0mmol/L. Nephropathy was defined as the estimated glomerular filtration rate (eGFR) < 60 mL/min/1.73 m2, and/or albuminuria. The eGFR was calculated using the Chronic Kidney Disease Epidemiology Collaboration (CKD-EPI) equation:

$$eGFR=141\times\min\left( \frac{Scr}{\kappa} , 1 \right)^{\alpha}\times\max\left( \frac{Scr}{\kappa} , 1 \right)^{-1.209}\times{0.993}^{Age}\times1.018 \left[ if female \right]\times1.159 [if non Hispanic black]$$

Where Scr is serum creatinine (mg/dL), κ is 0.7 for females and 0.9 for males, α is −0.329 for females and −0.411 for males, min indicates the minimum of Scr/κ or 1, and max indicates the maximum of Scr/κ or 1. Albuminuria was defined as an albumin-to-creatinine ratio (ACR) above 30 mg/g.

According to the PA Guidelines, physical activity (PA) was categorized as PA: adults engaged in ≥150 min/week of moderate-intensity PA, 75 min/week of vigorous-intensity PA, or an equivalent combination. We categorized PA as physically inactive participants (“not meeting PA Guidelines”) who did not meet 2018 PA Guidelines and physically active participants (“meeting PA Guidelines”) who met 2018 PA Guidelines). Hypertension was defined as meeting any of the following conditions: systolic blood pressure was greater than or equal to 140 mmHg, or diastolic blood pressure was greater than or equal to 90 mmHg, or they were currently taking medication to lower high blood pressure.

**Appendix 2**

**Supplementary Results**

| **Table S1** The lowest, 25^th^, 50^th^,75^th^ percentiles, and highest values of BPb, BCd, UPb, and UCd in NHANES 2007–2016 | | | | | | |
| --- | --- | --- | --- | --- | --- | --- |
| Metals exposure | LLOD^a^ | Lowest^b^ | 25^th^ | 50^th^ | 75^th^ | Highest |
| In blood | |  |  |  |  |  |
| BPb (ug/dL) | 0.070 | 0.050 | 0.870 | 1.280 | 2.000 | 25.000 |
| BCd (ug/L) | 0.100 | 0.070 | 0.220 | 0.350 | 0.590 | 8.800 |
| In urine | | | | | | |
| UPb (ug/L) | 0.030 | 0.020 | 0.260 | 0.450 | 0.800 | 13.400 |
| UCd (ug/L) | 0.036 | 0.030 | 0.150 | 0.290 | 0.520 | 6.700 |
| a. lower limit of detection (LLOD); | | | | | | |
| b. In cases, where the result was below the limit of detection, the value for that variable is the detection limit divided by the square root of two. | | | | | | |


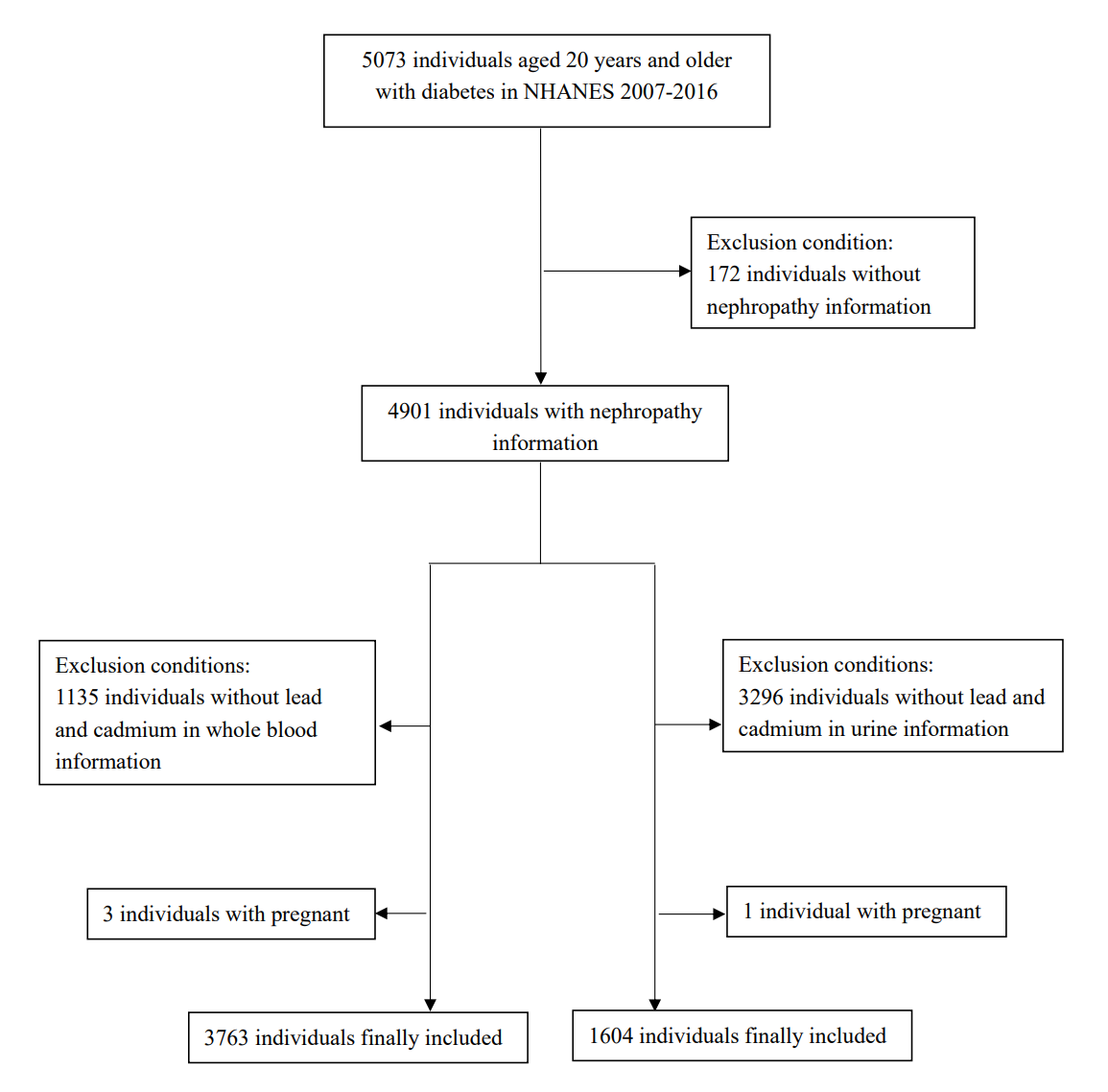


**Figure S1** Flow chart of the population included in the final analysis of our study. N (blood) =3763, N (urine) =1604, NHANES, the United States, 2007-2016
